# Development of Multiplex Real-Time Quantitative PCR for the Detection of *Giardia duodenalis*, *Enterocytozoon bieneusi*, and *Cryptosporidium* spp. in Dairy Goats

**DOI:** 10.3390/ani16060879

**Published:** 2026-03-11

**Authors:** Jing-Rui Liu, Xin Yang, Hao Chen, Xiao-Ying Zuo, Kai-Min Fang, Ying-Ying Fan, Wen-Pei Guo, Shi-Jie Dong, Guang-Hui Zhao, Jun-Ke Song

**Affiliations:** College of Veterinary Medicine, Northwest A&F University, Xianyang 712000, China; 17732563543@163.com (J.-R.L.); xinyang@nwafu.edu.cn (X.Y.);

**Keywords:** multiplex real-time quantitative PCR, *Taq*Man, zoonotic gastrointestinal protozoa

## Abstract

*Giardia duodenalis*, *Enterocytozoon bieneusi*, and *Cryptosporidium* spp. are common zoonotic intestinal protozoa in dairy goats, capable of cross-species transmission. These pathogens impair the growth and development of dairy goats and present a significant threat to public health. Conventional detection methods are time-consuming and lack sensitivity, requiring a rapid and efficient technique for simultaneous detection. A *Taq*Man probe-based multiplex real-time qPCR assay for the detection of the three protozoa was established in this study using specific primers and probes. Specificity validation demonstrated no cross-reactivity with other common intestinal pathogens of dairy goats. Sensitivity testing revealed minimum detection limits of 29.83, 39.33, and 33.15 copies/μL for the three protozoa, with excellent repeatability. Detection of clinical fecal samples and comparison with PCR confirmed the assay’s superior performance, which was capable of identifying positive samples missed by PCR. In conclusion, this rapid, specific, and sensitive assay provides technical support for large-scale epidemiological surveys and real-time monitoring of related protozoan diseases in dairy goats, thereby laying a scientific foundation for prevention and control and safeguarding both the dairy goat industry and public health.

## Simple Summary

In this study, a *Taq*Man probe-based multiplex real-time qPCR assay was established for the simultaneous detection of three common zoonotic intestinal protozoa in dairy goats: *Giardia duodenalis*, *Enterocytozoon bieneusi*, and *Cryptosporidium* spp. The assay was designed using specific primers and probes, and specificity validation confirmed no cross-reactivity with other common intestinal pathogens in dairy goats. Sensitivity testing revealed minimum detection limits of 29.83, 39.33, and 33.15 copies/μL for the three protozoa, with excellent repeatability. Detection of clinical fecal samples, compared with PCR, demonstrated the assay’s superior performance, capable of identifying positive samples missed by PCR. In conclusion, this rapid, specific, and sensitive assay provides technical support for large-scale epidemiological surveys and real-time monitoring of related protozoan diseases in dairy goats, thus laying a scientific foundation for prevention and control and safeguarding both the dairy goat industry and public health.

## 1. Introduction

Dairy goats are a crucial part of the global dairy industry, providing nutrient-rich products that are essential for human diets worldwide [1]. However, these animals are highly susceptible to gastrointestinal protozoan infections, with *Giardia duodenalis*, *Enterocytozoon bieneusi*, and *Cryptosporidium* spp. posing substantial threats to both animal health and public safety. As typical zoonotic pathogens, these protozoa exhibit broad host ranges, strong infectivity, and widespread distribution, capable of inducing severe gastrointestinal disorders in both dairy goats and humans [2]. Infected dairy goats excrete large quantities of infectious propagules, including *Cryptosporidium* spp. oocysts, *E. bieneusi* spores, and *Giardia duodenalis* cysts via feces [3,4,5,6]. These pathogens can contaminate the surrounding environment, feed, and water sources as well as further contaminate dairy products during production processes [7]. The consumption of contaminated dairy products constitutes a direct route for human infection, thereby imposing a significant burden on public health.

*Cryptosporidium* spp. are obligate intracellular parasites that infect both humans and a wide range of animals [8]. Infection in dairy goats typically causes acute or chronic diarrhea, which can be life-threatening in severe cases. In addition to animals, immunocompromised individuals, including AIDS patients and those undergoing immunosuppressive therapy, are also susceptible to infection [9,10,11]. *Giardia duodenalis,* a flagellated protozoan, colonizes the host’s small intestine by adhering to the intestinal mucosa via its adhesive disc, triggering symptoms like abdominal pain, chronic diarrhea, nausea, and malabsorption [12,13,14]. This pathogen is transmitted primarily through the fecal–oral route and can survive in the environment for extended periods, making it highly transmissible in areas with poor sanitation [15,16]. *Enterocytozoon bieneusi,* a microsporidian parasite, is one of the most common species associated with microsporidiosis in both animals and humans [17,18]. It invades enterocytes and replicates within host cells, causing various gastrointestinal symptoms [19]. Infected animals may exhibit persistent and severe clinical signs, whereas immunocompromised individuals are also at risk of infection. This parasite is primarily transmitted via the fecal–oral route through contaminated water and food sources [20,21].

Traditional detection methods for these protozoa have inherent limitations, which hinder efficient surveillance and diagnosis. Microscopic examination heavily relies on the expertise of technicians and lacks sufficient sensitivity to identify low-level infections. Serological assays, while capable of detecting pathogen-specific antibodies, cannot provide quantitative data on the burden of pathogens [22]. Although molecular techniques, such as polymerase chain reaction (PCR), have become the gold standard for protozoan detection due to their high sensitivity and specificity [23], most existing PCR-based methods target only one or a limited number of pathogens. This makes the simultaneous screening of multiple protozoa time-consuming, which is unsuitable for large-scale epidemiological investigations [24]. Real-time quantitative PCR (qPCR), combined with *Taq*Man probe technology, enables rapid, specific, and quantitative detection of pathogens, while multiplex qPCR further allows the simultaneous identification of multiple targets in a single reaction [25,26]. However, to date, no *Taq*Man multiplex qPCR assay has been developed for the simultaneous detection of *G. duodenalis*, *E. bieneusi*, and *Cryptosporidium* spp. in dairy goats.

Therefore, this study aims to develop and validate a *Taq*Man multiplex qPCR assay for the simultaneous detection of the three zoonotic protozoa in dairy goats. The establishment of this method is expected to provide a rapid and reliable tool for epidemiological surveys of the three protozoa in dairy goats, thereby supporting the prevention and control of zoonotic diseases and ensuring the safety of dairy products.

## 2. Materials and Methods

### 2.1. Ethical Approval

All procedures involving animal sample collection were conducted in accordance with the ARRIVE guidelines and the Chinese national standard GB/T 35892-2018 [27,28]. Ethical approval was not required as only non-invasive fecal anal swabs were collected without restraint or tissue injury, which are exempted under Article 4 of GB/T 35892-2018 for epidemiological surveillance. Samples were collected by licensed veterinarians with permission from the Dairy Goats Breeding Center in Heshui County, Gansu Province, China. Sterile soft swabs were used to minimize animal stress, and no adverse reactions were observed during or after the sampling process.

### 2.2. Sample Collection

A total of 142 fecal samples were selected based on the breeding scale and pen distribution at the Heshui Dairy Goats Breeding Center. The farm has a total of 3000 lambs, with an overall inventory of 5800. The young dairy goats (under 6 months of age) were selected as the subjects for clinical sample collection in this experiment. To ensure the balance of sampling and minimize experimental errors caused by pen conditions and lamb health status, the 142 samples were collected evenly from 14 different lamb pens, with an average of 10 samples collected from each pen. The samples were placed in an icebox marked with basic information and stored at 4 °C until required.

### 2.3. DNA Extraction

DNA samples were extracted according to the instructions of the E.Z.N.A. Stool DNA Kit (Omega Bio-tek, Inc, Norcross, GA, USA). DNA from the three target protozoa was verified as positive using the published primers in Table 1 [29] and then stored at −20 °C for use in subsequent experiments. Specific parasite DNA samples, preserved in the Veterinary Parasitology Laboratory, were stored at −80 °C for long-term use in future research.

### 2.4. Design and Synthesis of Specific Primers and Probes

Primers and *Taq*Man probes for the detection of *Cryptosporidium* spp., *Giardia duodenalis* and *Enterocytozoon bieneusi* were designed using Primer Premier 6.0 and Beacon Designer 8.0. The selected gene loci include *18S* of *E. bieneusi*, *HSP70* of *Cryptosporidium* spp., and *gdh* of *Giardia duodenalis*, respectively. The specificity of the three pairs of primers and probes was then verified using the Blastn search engine (http://www.ncbi.nlm.nih.gov/ (accessed on 10 September 2025)). The primers and probes used in this study were synthesized by Bioengineering Company (Table 2). The 5′ end and 3′end of the *Taq*Man probe used in this study were labeled with the FAM, Cy5, and VIC fluorophores, respectively.

### 2.5. Plasmid Construction and Standard Curve Generation

Total DNA of *Cryptosporidium* spp., *Giardia duodenalis* and *Enterocytozoon bieneusi* was extracted as templates and verified using the primers in Table 1. The corresponding genes of the three species were amplified by conventional PCR. After the amplification, the target amplification bands were confirmed by gel electrophoresis. Subsequently, the gel products were recovered and ligated into the pMD19-T vector, and the standard recombinant plasmid was constructed and named accordingly. The plasmid concentrations were quantified using a spectrophotometer (Berthold, Wildbad, Germany), and the number of copies was calculated using the following formula: C (copies/µL) = (6.02 × 10^23^) × (c (ng/µL) × 10^−9^ DNA)/(DNA length × 660). To avoid the competitive effects between different plasmids leading to deviations in the standard curve, the standard plasmids were diluted separately using single-target plasmids for the generation of standard curves. The serially diluted standard plasmids were amplified using the optimized *Taq*Man qPCR system and reaction conditions. The final standard curve was generated based on the Ct value and the logarithm of the standard copy number.

### 2.6. TaqMan Multiplex Quantitative PCR

*Taq* Pro U+ Multiple Probe qPCR Mix (Vazyme, Xi’an, China) and a qPCR system (Tianlong Technology Co., Ltd., Xi’an, China) were used for this qPCR assay. The concentrations of the primers and probes in the qPCR were optimized (Tables S1 and S2) to achieve the maximal cycle-to-cycle fluorescence increase (ΔRn) for each distinct fluorescent signal and the lowest possible threshold cycle (Ct). The concentrations of both primers and probes used in this method were 0.01 nmol/μL. The reaction setup included 2 μL of standard DNA, 10 μL of Probe qPCR Mix, three pairs of qPCR primers (*Enterocytozoon bieneusi-*F/R, *Cryptosporidium* spp.-F/R, and *Giardia duodenalis*-F/R), three pairs of TaqMan probes (Table 2), and 4.4 μL of nuclease-free water, resulting in a final reaction volume of 20 μL The reaction conditions, including various annealing temperatures and the number of amplification cycles, were optimized. The amplification process was executed using the following thermal cycling parameters: 37 °C for contamination digestion for 2 min, 95 °C for predenaturation for 30 s, 95 °C for denaturation for 10 s, 60 °C for annealing for 30 s, and a total of 40 cycles. The real-time fluorescence intensities were measured at the end of each annealing step. After the analysis of each sample, the Ct values were determined by using the log-linear phase of each individual reaction. Positive and negative controls were included in all reactions to ensure the reliability and accuracy of the assay.

### 2.7. Specificity, Sensitivity and Repeatability

To validate the specificity, a DNA mixture of *Cryptosporidium* spp., *Giardia duodenalis* and *Enterocytozoon bieneusi* was used as the positive template, genomic DNA of seven common intestinal pathogens of dairy goats (*Eimeria* spp., *Haemonchus contortus*, *Moniezia* spp., *Oesophagostomum asperum*, *Listeria monocytogenes*, *Escherichia coli* and *Staphylococcus aureus*) as non-target templates, and nuclease-free water as the negative control. The multiplex real-time quantitative PCR assay established in this study was performed with three technical replicates to verify the absence of cross-reactivity. Meanwhile, the corresponding singleplex PCR was conducted in parallel for all the above samples, with the single-target PCR results serving as the gold standard (samples with positive amplification products were considered positive). Furthermore, the concordance of detection results between the two assays was analyzed and validated.

To validate the sensitivity of this method, standard plasmids of *Cryptosporidium* spp., *Giardia duodenalis* and *Enterocytozoon bieneusi,* with concentrations of 2.983 × 10^10^ copies to 2.983 × 10^1^ copies, 3.933 × 10^10^ copies to 3.933 × 10^1^ copies and 3.315 × 10^10^ copies to 3.315 × 10^1^ copies respectively, were used as positive control templates for *Taq*Man qPCR amplification, and ddH_2_O was set as the negative control. The minimum detection limit (MDL) of this method for three types of pathogens were analyzed through amplification curves and Ct values. The minimum detection limit (MDL) of the assay was defined as the lowest concentration with a positive detection rate ≥95%, as determined through amplification curves and Ct values. Single-target PCR results were also used as the gold standard to further evaluate the assay’s sensitivity.

To validate the assay repeatability, the standard plasmids of *Cryptosporidium spp*., *Giardia duodenalis* and *Enterocytozoon bieneusi* (2.983 × 10^5^–2.983 × 10^3^, 3.933 × 10^5^–3.933 × 10^3^ and 3.315 × 10^5^–3.315 × 10^3^ copies/μL, respectively) were used as positive controls and subjected to three replicate amplifications per concentration, with ddH_2_O serving as the negative control. Each concentration was tested three times to ensure the reliability of the results. The average Ct value was calculated based on the results, and the repeatability of this method was evaluated using the coefficient of variation (CV) formula.

### 2.8. Establishment of Standard Curves for Cryptosporidium spp., Giardia duodenalis and Enterocytozoon bieneusi

Standard plasmids of *Cryptosporidium* spp., *Giardia duodenalis* and *E. bieneusi*, with concentrations ranging from 2.983 × 10^10^ copies to 2.983 × 10^1^ copies, 3.933 × 10^10^ copies to 3.933 × 10^1^ copies and 3.315 × 10^10^ copies to 3.315 × 10^1^ copies, respectively, were used as templates, with ddH_2_O serving as the negative control. Three replicate amplifications were performed using the optimized reaction system and conditions, and the standard curve was constructed based on the average Ct values.

### 2.9. Detection of Clinical Samples

A total of 142 fecal samples were tested using the established multiplex qPCR assay and conventional PCR for comparison. The three pathogens in the 142 collected goat fecal samples were detected using the method established in this study and conventional PCR, and the detection results of both methods were compared. The primer sequences used in the conventional PCR method for comparison in this study were derived from published research by Wang Junwei et al. [29].

### 2.10. Statistical Analysis

Data analysis was performed using SPSS 26.0 software. The chi-square test was used to compare the detection rates of the three parasites (*Enterocytozoon bieneusi*, *Cryptosporidium* spp., and *Giardia duodenalis*) and mixed infections in 142 clinical samples between the established multiplex real-time quantitative PCR assay and conventional PCR. A *p*-value < 0.05 was considered statistically significant. Additionally, the Kappa coefficient was calculated to evaluate the consistency between the two methods (Kappa value range: 0–1, with higher values indicating better agreement).

## 3. Results

### 3.1. Optimization of Reaction Conditions and Reaction System for the TaqMan Multiplex Real-Time Quantitative PCR Assay

**Figure 1:**
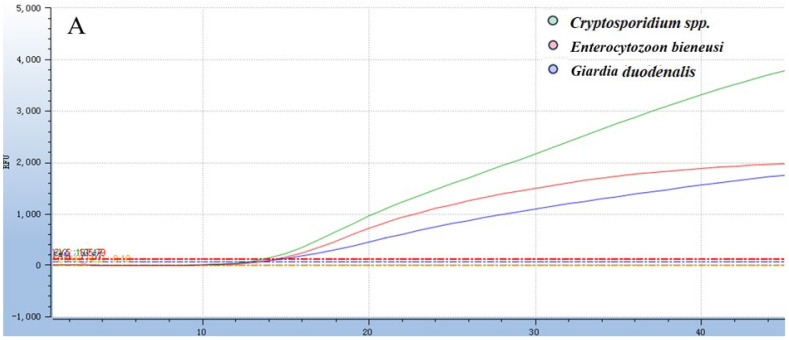

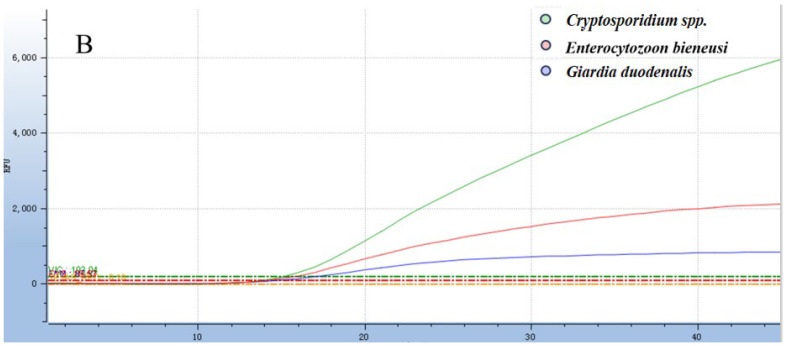
Optimization of reaction conditions for the *Taq*Man multiplex real-time quantitative PCR assay. (**A**) Optimal primer concentrations for *Taq*Man multiplex fluorescence qPCR. (**B**) Optimal probe concentrations for *Taq*Man multiplex fluorescence qPCR.

**Figure 2:**
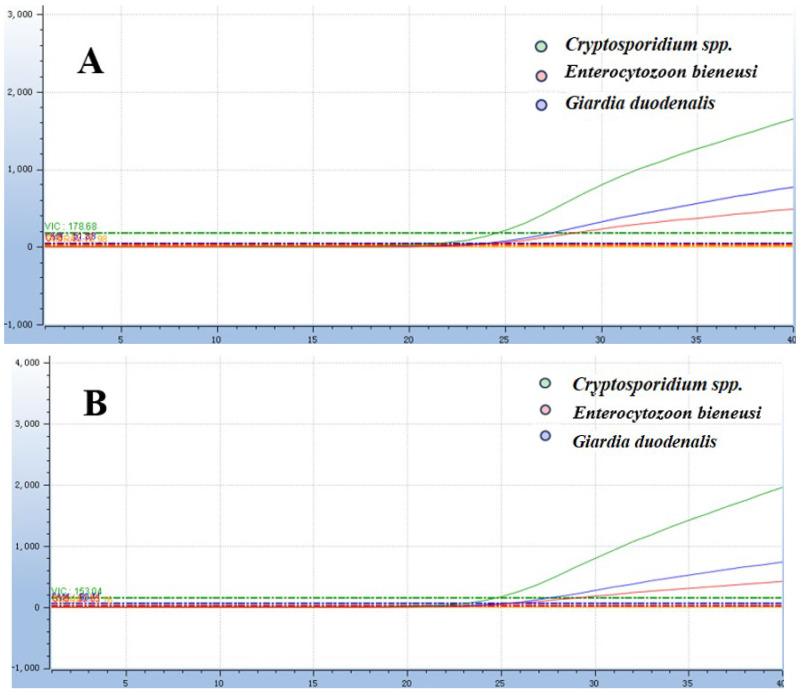

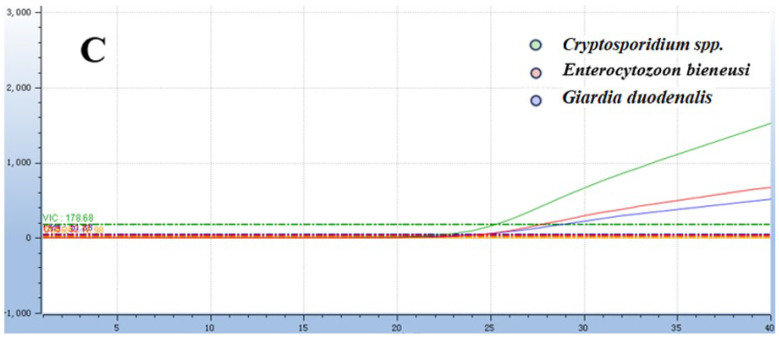
Optimization of annealing temperature for the *Taq*Man multiplex fluorescence qPCR assay. (**A**) The annealing temperature is 60 °C. (**B**) The annealing temperature is 62 °C. (**C**) The annealing temperature is 64 °C.

The optimal reaction system was obtained by optimizing each reaction condition using the matrix method (the concentrations of both primers and probes used in this method were 0.01 nmol/μL): 0.004 nmol of each of the F/R primer for *Cryptosporidium* spp., 0.006 nmol of the probe, 0.006 nmol of each of the F/R primer for *Giardia duodenalis,* 0.006 nmol of the probe, 0.006 nmol of each of the F/R primer for *Enterocytozoon bieneusi*, 0.002 nmol of the probe, 3.0 μL of a mixture of three recombinant plasmid standards, ddH_2_O was added to reach a final reaction volume of 20 μL (Figure 1 and Figure 2). The amplification process was executed using the following thermal cycling parameters: 37 °C for contamination digestion for 2 min, 95 °C for predenaturation for 30 s, 95 °C for denaturation for 10 s, 60 °C for annealing for 30 s, and a total of 40 cycles.

### 3.2. Specificity of the TaqMan Multiplex Real-Time Quantitative PCR Assay

**Figure 3:**
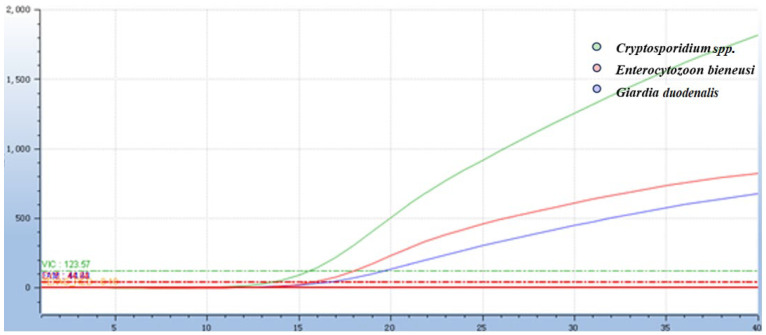
Specificity test of the *Taq*Man multiplex real-time quantitative PCR assay. The DNA of other pathogens unrelated to *Cryptosporidium* spp., *Giardia,* and *E. bieneusi* was used as a template. Other pathogens include *Eimeria*; *Moniezia*; *H. contortus*; *O. asperum*; *L. monocytogenes*; *E. coli*; and *S. aureus*. The results showed that fluorescence signals could be detected for the three pathogens labeled with VIC (*Cryptosporidium* spp.), FAM (*Giardia duodenalis*), and CY5 (*E. bieneusi*) respectively. However, for the other pathogens, there was no amplification curve, which was the same as that of the negative control.

The results showed that the fluorescence signals of the three pathogens, including FAM, VIC, and CY5, could all be detected (Figure 3). For the negative control and other unrelated pathogens (*Eimeria*, *Haemonchus contortus*, *Moniezia*, *Oesophagostomum asperum*, *Escherichia coli* etc.), no specific fluorescent signals were detected in the assay, indicating the assay had 100% specificity. Retesting of the positive samples, non-target pathogens and negative controls identified by the multiplex real-time qPCR was performed using single-target PCR [29], with all results showing complete concordance with the established assay (Table 3). No false positive results were observed, which further verified the assay’s 100% specificity.

### 3.3. Sensitivity of the TaqMan Multiplex Real-Time Quantitative PCR Assay

**Figure 4:**
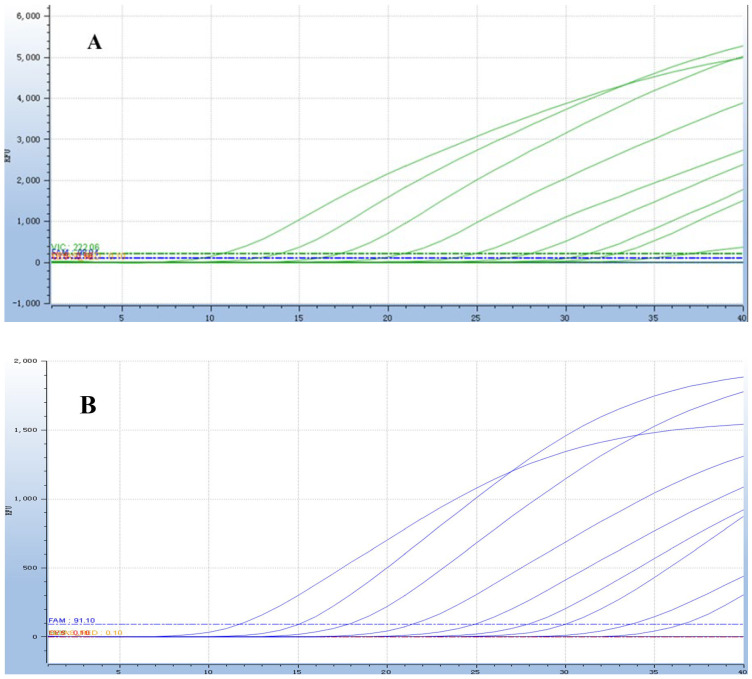

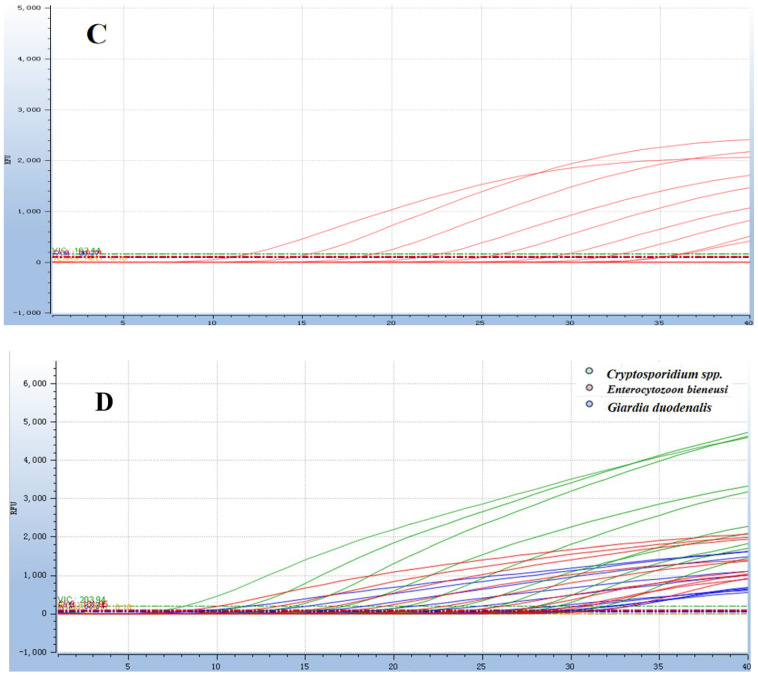
Sensitivity test of the *Taq*Man multiplex real-time quantitative PCR assay. (**A**) The results of the sensitivity amplification curves of *Cryptosporidium* spp.; the plasmid concentrations of *Cryptosporidium* spp. were 2.983 × 10^10^ copies to 2.983 × 10^1^ copies. (**B**) The results of the sensitivity amplification curves of *Giardia*; the plasmid concentrations of *Giardia duodenalis* were 3.933 × 10^10^ copies to 3.933 × 10^1^ copies. (**C**) The results of the sensitivity amplification curves of *E. bieneusi*; the plasmid concentrations of *E. bieneusi* were 3.315 × 10^10^ copies to 3.315 × 10^1^ copies. (**D**) The results of the sensitivity amplification curves of three pathogens, and a negative control was set up in each group of the assay.

The results showed that all three target protozoa presented typical concentration-dependent amplification curves, and the fluorescent signal intensity decreased gradually with the reduction in plasmid concentration (Figure 4). The MDLs of the assay for *Cryptosporidium spp*., *Giardia duodenalis* and *Enterocytozoon bieneusi* were 29.83, 39.33 and 33.15 copies/μL, respectively. The further diluted concentration showed negative results in all three repetitions, confirming the accuracy of the minimum detection limit. Subsequent clinical sample detection using conventional PCR further verified that the detection limit of the established assay was much lower than that of conventional PCR. All positive samples detected by conventional PCR were identified by the qPCR assay, with no false negatives, confirming 100% diagnostic sensitivity of the assay within the detection range of the gold standard. Additional positive samples detected only by qPCR were verified as true positives of the target protozoa by Sanger sequencing, directly demonstrating the high sensitivity advantage of the established assay. Statistical analysis results of the two methods are detailed in Section 3.6.

### 3.4. Repeatability of the TaqMan Multiplex Real-Time Quantitative PCR Assay

Using a 10-fold serial dilution (ranging from 10^3^ to 10^5^ copies) of plasmid standard mixtures as templates, the intra- and inter-assay repeatability of the *Taq*Man multiplex quantitative PCR method developed in this study was evaluated. The results showed that the coefficient of variation (CV) was less than 3.0% (Table 4). This indicates that the method has good repeatability.

### 3.5. Standard Curves for the TaqMan Multiplex Real-Time Quantitative PCR Assay

**Figure 5:**
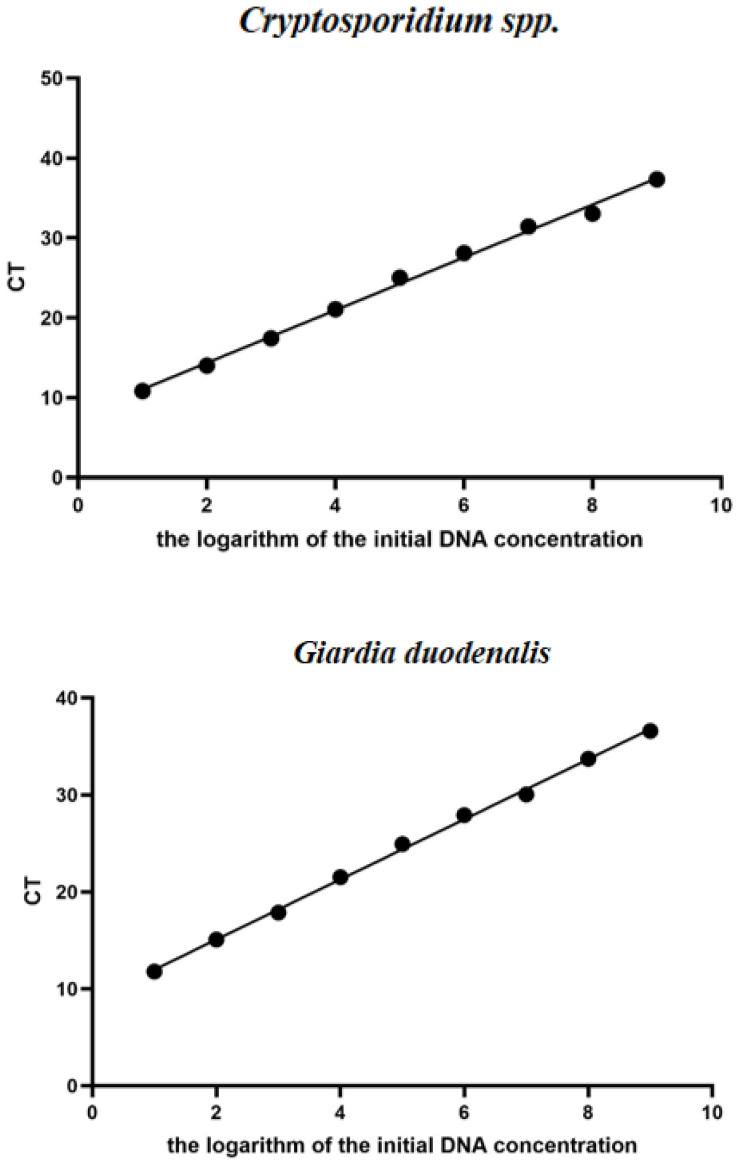

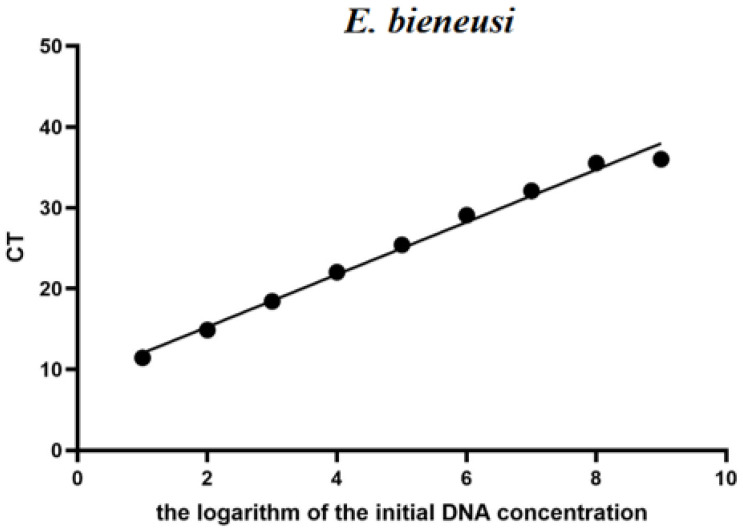
Standard curves for the *Taq*Man multiplex real-time quantitative PCR assay. The quantified DNA plasmids of *Cryptosporidium* spp., *Giardia duodenalis* and *E. bieneusi* with concentrations of 2.983 × 10^10^ copies to 2.983 × 10^1^ copies, 3.933 × 10^10^ copies to 3.933 × 10^1^ copies and 3.315 × 10^10^ copies to 3.315 × 10^1^ copies respectively, employed as a positive control, were diluted three times in 10-fold serial dilutions, and the examination of these three replicates produced the curve.

The optimized conditions were applied for amplification, and the standard curve was constructed using the Ct values and logarithm of DNA copy number obtained by the DNA gradient multiplier dilution of the standard plasmids of *Cryptosporidium* spp., *Giardia,* and *E. bieneusi* prepared in this study. The results showed that the correlation coefficients R^2^ were 0.9959, 0.9982 and 0.9901, respectively. The standard formulas are y = 3.3028x + 7.7481, y = 3.0987x + 8.8956, and y = 3.2433x + 8.7800, respectively (Figure 5).

### 3.6. Clinical Performance and Statistical Comparison

**Figure 6:**
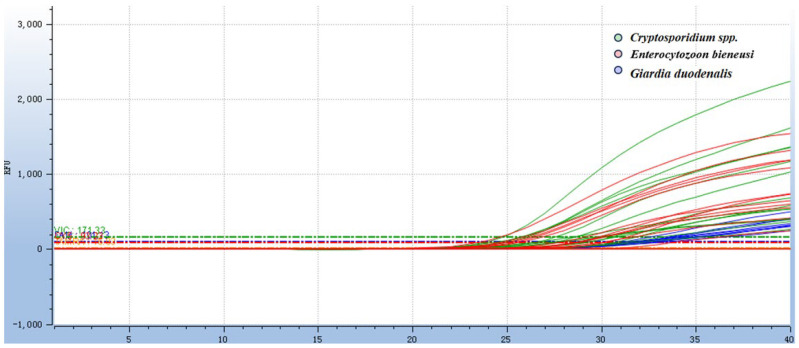
The result of partial clinical samples test of multiplex *Taq*Man qPCR for three zoonotic gastrointestinal protozoa in dairy goats.

**Figure 7:**
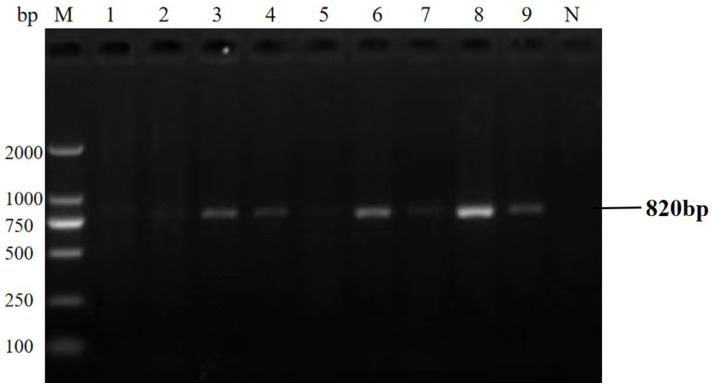
The result of partial clinical samples test of PCR for *Cryptosporidium* spp. M: 2000 bp DNA marker, 1–9: partial clinical DNA sample PCR test results, N: negative control.

**Figure 8:**
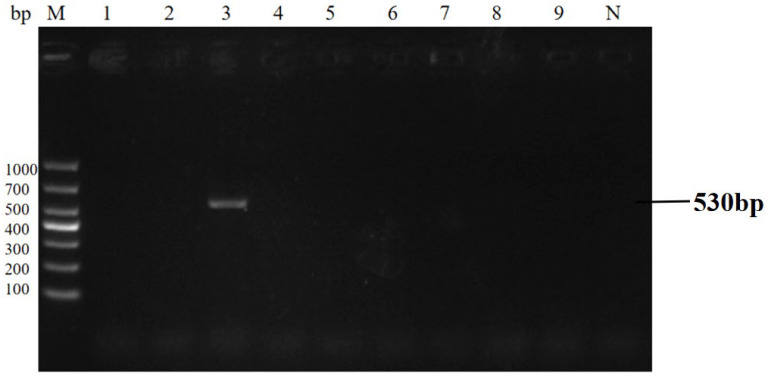
The result of partial clinical samples test of PCR for *Giardia duodenalis*. M: 1000 bp DNA marker, 1–9: partial clinical DNA sample PCR test results, N: negative control.

**Figure 9:**
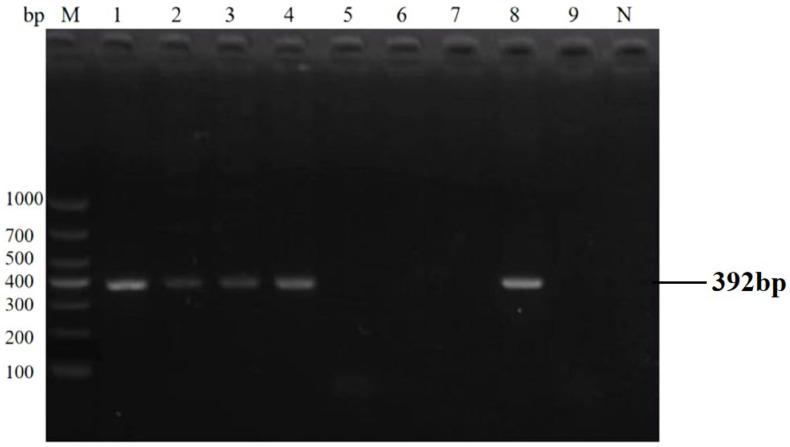
The result of partial clinical samples test of PCR for *E. bieneusi*. M: 1000 bp DNA marker, 1–9: partial clinical DNA sample PCR test results, N: negative control.

The qPCR assay identified 40 positive samples for *E. bieneusi*, 25 for *Cryptosporidium* spp., 27 for *Giardia duodenalis*, and 38 for mixed infections (Table 5). Statistical analysis (χ^2^ test) revealed a significant difference in detection rates between the two methods (*p* < 0.05). The qPCR assay showed 100% concordance with the gold standard, confirming its superior detection rate and its ability to detect all positive cases without missing any. The concordance rate results also indicate that the sensitivity of the qPCR method is 100%. Detection results of partial clinical samples are shown in Figure 6, Figure 7, Figure 8 and Figure 9. The overall detection rate of multiplex qPCR (26.76%) was significantly higher than conventional PCR (9.86%), as detailed in Table 5. Consistency analysis showed a Kappa value of 0.62 (95% CI: 0.51–0.73), indicating moderate agreement between the two methods. However, multiplex qPCR demonstrated better performance in detecting low-abundance and mixed infections.

## 4. Discussion

Zoonotic gastrointestinal protozoosis in dairy goats is caused by various gastrointestinal protozoa, primarily transmitted via the fecal–oral route. This disease can affect goats of various breeds and ages; however, young goats, particularly those aged 1 to 3 months, exhibit higher incidence and mortality rates due to their developing immune systems. Currently, zoonotic gastrointestinal protozoosis in dairy goats significantly impacts production traits such as milk and meat yield, leading to substantial global economic losses. It has emerged as a major challenge for the goat farming industry.

In this study, a multiplex *Taq*Man fluorescence quantitative PCR assay targeting the *hsp70, gdh,* and *18S* regions was successfully established for detecting *Cryptosporidium* spp., *Giardia duodenalis*, and *Enterocytozoon bieneusi* in goats. The *18S rRNA* gene of *E. bieneusi* is highly conserved but contains variable regions, which have been widely used in phylogenetic and taxonomic studies of protozoa. For *Cryptosporidium* spp., the *HSP70* gene has been shown to be useful for species identification and phylogenetic analysis. For example, studies on the 70 kDa heat shock protein genes of various *Cryptosporidium* species have shown its value in differentiating species and genotypes within the genus [30,31]. The *gdh* gene of *Giardia duodenalis* has been proven to be a reliable target for genotyping and detection. PCR-RFLP targeting the *gdh* locus has been successfully used to identify *Giardia duodenalis* isolates directly from feces [32]. Through systematic optimization of primer–probe combinations and reaction conditions, the assay achieved highly specific and multiplex detection of these clinically relevant protozoa. The sensitivity of the assay for the three protozoa has the lowest detection limits, reaching 29.83 copies/μL for *Cryptosporidium* spp., 39.33 copies/μL for *Giardia duodenalis*, and 33.15 copies/μL for *Enterocytozoon bieneusi*, with a 100% detection rate within the assay range. The assay demonstrated excellent repeatability, with coefficients of variation (CV) below 3%, and showed no cross-reactivity with common intestinal parasites. Based on the comparison with conventional PCR coupled with DNA sequencing, the sensitivity and specificity of the established multiplex qPCR assay were both 100% for the three protozoan pathogens. When applied to clinical samples, the detection performance of this assay far surpassed that of traditional methods.

Detection of zoonotic gastrointestinal protozoa in dairy goats has historically relied on various traditional and molecular methods, each with limitations that hinder efficient and accurate surveillance. Numerous pathogens cause diarrhea in goats in clinical settings, making it challenging to determine if the cause is a zoonotic intestinal protozoan infection based on clinical symptoms alone. Traditional morphological detection methods, such as microscopic examination of fecal samples, are routinely used to detect parasites; however, they struggle to detect latent infections and are highly dependent on the expertise of technicians to identify protozoan stages (oocysts, cysts, or spores) [33,34]. Accurate diagnosis of this disease, particularly during its latent period or prodromal stage, ultimately requires laboratory confirmation [35]. Serological methods, typically used to detect host antibodies against protozoan antigen, are prone to cross-reactivity with related pathogens, leading to false positives, and cannot provide quantitative data. Additionally, antibody detection reflects past or current exposure rather than active infection, limiting its utility for real-time surveillance. Molecular biology methods are one of the main means of laboratory diagnosis. Currently, polymerase chain reaction (PCR) is the most widely used detection method, which has revolutionized protozoan detection by overcoming the sensitivity limitations of morphological and serological methods; however, it is also prone to false positive results during the detection process [36]. Most existing PCR-based assays are designed for single or limited pathogen targets [37]. Compared with the *COWP* gene-based qPCR for *Cryptosporidium* spp. [38], *Taq*Man qPCR showed far higher sensitivity [39]. The probe-based, closed-tube system also avoids non-specific amplification from SYBR Green and nucleic acid cross-contamination, offering higher detection efficiency. Single-target designs cannot meet the demand for simultaneous screening of multiple pathogen co-infections in dairy goats. Despite its potential, no previous studies have applied this technology to simultaneously detect *Cryptosporidium* spp., *Giardia duodenalis,* and *Enterocytozoon bieneusi*. However, multiplex PCR assays for these pathogens have also been reported in recent years. [40] successfully developed a multiplex PCR system for the simultaneous detection of *G. duodenalis*, *C. parvum*, *Blastocystis* spp. and *E. bieneusi* in goat fecal samples, which achieved 100% sensitivity and specificity with a minimum detection limit of ≥10^2^ copies/μL. The assay established in this study also exhibited 100% sensitivity and specificity for *G. duodenalis*, *Cryptosporidium* spp. and *E. bieneusi*, with a significantly lower limit of detection. Furthermore, unlike the conventional multiplex PCR assay by Yu et al., which relied on agarose gel electrophoresis for result interpretation, the present assay enables quantitative detection of target pathogens without post-PCR electrophoresis. This shortens the overall detection cycle and reduces the risk of nucleic acid cross-contamination caused by open-tube manipulation. The successful establishment of this multiplex *Taq*Man fluorescence quantitative PCR assay provides valuable support for the molecular epidemiological investigation of zoonotic gastrointestinal protozoan infections in goats, facilitating more effective prevention and control strategies.

## 5. Conclusions

In conclusion, this study has successfully established a multiplex real-time quantitative PCR assay for the simultaneous detection of *Giardia duodenalis*, *Enterocytozoon bieneusi* and *Cryptosporidium* spp. in dairy goats. The assay demonstrates high sensitivity, specificity, and repeatability, providing a reliable, rapid, and quantitative method for detecting these zoonotic gastrointestinal protozoa. The results provide a theoretical basis for the efficient prevention and control of dairy goat parasitosis, helping assess the risk of environmental contamination by these pathogens and ensuring public health safety.

## Figures and Tables

**Table 1:** Published primers used for verification of positive DNA samples of target protozoa.

Species	Gene Loci	Primer Name	Primer Sequence (5′→3′)	Product Size (bp)
*E*. *bieneusi*	*ITS rRNA*	F1	GGTCATAGGGATGAAGAG	410
		R1	TTCGAGTTCTTTCGCGCTC	
		F2	GCTCTGAATATCTATGGCT	392
		R2	ATCGCCGACGGATCCAAGTG	
*Giardia duodenalis*	*gdh*	*gdh*1	TTCCGTRTYCAGTACAACT	754
		*gdh*2	ACCTCGTTCTGRGTGGCGCA	
		*gdh*3	ATGACYGAGCTYCAGAGGCACGT	530
		*gdh*4	GTGGCGCARGGCATGATGCA	
*Cryptosporidium* spp.	*18S rRNA*	*18S* F1	TTCTAGAGCTAATACATGCG	1325
		*18S* R1	CCCATTTCCTTCGAAACAGGA	
		*18S* F2	GGAAGGGTTGTATTTATTAGATAAAG	820
		*18S* R2	AAGGAGTAAGGAACAACCTCCA	

**Table 2:** Primers and probes used in the *Taq*Man qPCR.

Species	Primer Name	Primer Sequence (5′→3′)	Product Size (bp)
*Enterocytozoon bieneusi*	E-*18S*-F	GCGGTAACTCCAACTCCAAGA	238
	E-*18S*-R	GATACTCCTGCCTTCGTCCTT	
	E-*18S*-P	CY5-TGTCGCCTTCGCCTCCGTTAGT-BHQ2	
*Cryptosporidium* spp.	C-*HSP70*-F	TCTACTGATGGCAACTGAAT	203
	C-*HSP70*-R	TTGATAACAGACTCGTGGAA	
	C-*HSP70*-P	VIC-CACATTGAGTTCTGAGTC-BHQ1	
*Giardia duodenalis*	G-*gdh*-F	GCCTCTGTCAATCAAGGG	186
	G-*gdh*-R	CTCCGTCAATGCCTTCAA	
	G-*gdh*-P	FAM-AGAAGGCGATCAGACACCACC-BHQ1	

Note: Specific primers and probes were designed by comparing the sequences of three pathogens deposited in GenBank. The gene-specific probe for *Enterocytozoon bieneusi* was labeled with CY5 at the 5’ end, the gene-specific probe for *Cryptosporidium* spp. was labeled with VIC, and the gene-specific probe for *Giardia duodenalis* was labeled with FAM, enabling the specific multiplex detection of *Enterocytozoon bieneusi*, *Cryptosporidium* spp., and *Giardia duodenalis* in a single reaction. By analysis of published nucleotide sequence data, the matching rates of the primers and probes for *Enterocytozoon bieneusi*, *Cryptosporidium* spp. and *Giardia duodenalis* were found to be 100%.

**Table 3:** Specificity verification results with PCR.

Parasite	True Positive	False Positive	True Negative	False Negative	Specificity (%)	Positive Predictive Value (%)	Negative Predictive Value (%)
*Cryptosporidium* spp.	3	0	24	0	100	100	100
*G. duodenalis*	3	0	24	0	100	100	100
*E. bieneusi*	3	0	24	0	100	100	100

**Table 4:** Intra-assay and inter-assay variation in *Taq*Man multiplex fluorescent qPCR assay of *Cryptosporidium* spp., *Giardia*, and *E. bieneusi*.

Plasmid	Plasmid Concentration (Copies/μL)	Intra-Assay Variability Test		Inter-Assay Variability Test	
		Mean ± SD	CV (%)	Mean ± SD	CV (%)
*E. bieneusi*	3.315 × 10^3^	15.70 ± 0.46	2.92	15.94 ± 0.42	2.63
	3.315 × 10^4^	18.11 ± 0.32	1.77	18.26 ± 0.35	1.91
	3.315 × 10^5^	21.55 ± 0.35	1.62	21.54 ± 0.38	1.76
*G. duodenalis*	3.933 × 10^3^	15.76 ± 0.44	2.79	15.85 ± 0.26	1.64
	3.933 × 10^4^	18.14 ± 0.34	1.87	18.24 ± 0.35	1.91
	3.933 × 10^5^	21.61 ± 0.28	1.29	21.50 ± 0.34	1.58
*Cryptosporidium* spp.	2.983 × 10^3^	15.49 ± 0.35	2.26	15.88 ± 0.39	2.46
	2.983 × 10^4^	18.06 ± 0.34	1.88	18.24 ± 0.35	1.92
	2.983 × 10^5^	21.46 ± 0.33	1.54	21.51 ± 0.35	1.63

Note: Intra-assay variation: For each concentration, triple replicates were set up on a single plate; the CV% (threshold ≤ 3%) and Ct values for each concentration are presented. Inter-assay variation: In this study, the multiplex qPCR was repeated on days 1, 2 and 3; the CV% (threshold ≤ 3%) and Ct values for each concentration are presented.

**Table 5:** The results and statistical comparison of clinical samples for three zoonotic gastrointestinal protozoa.

Species	No. Positive (%, 95% CI)		χ^2^ Value	df	*p*-Value	Concordance Rate (%)
	PCR	*Taq*Man qPCR				
*E. bieneusi*	31 (21.83, 15.03–28.63)	40 (28.17, 20.98–35.96)	4.28	1	0.039	100
*Cryptosporidium* spp.	16 (11.27, 6.58–16.56)	25 (17.61, 11.89–23.33)	5.12	1	0.024	100
*Giardia duodenalis*	2 (1.41, 0.17–2.65)	27 (19.01, 13.09–24.93)	28.36	1	<0.001	100
Mixed infection	14 (9.86, 5.59–14.13)	38 (26.76, 19.68–33.84)	12.75	1	<0.001	100

## Data Availability

The original contributions presented in this study are included in the article/Supplementary Materials. Further inquiries can be directed to the corresponding authors.

## Supplementary Materials

The following supporting information can be downloaded at: https://www.mdpi.com/article/10.3390/ani16060879/s1, Supplementary Table S1. Square table of primer reaction quantities of three species. Supplementary Table S2. Square table of probe reaction quantities of three species.
