# Supplementary material for: Development of Multiplex Real-Time Quantitative PCR for the Detection of Giardia duodenalis, Enterocytozoon bieneusi, and Cryptosporidium spp. in Dairy Goats
Source: Animals (Basel). 2026 Mar 11;16(6):879. doi: 10.3390/ani16060879 (PMC13023305; doi:10.3390/ani16060879)
Supplement: Supplementary file 1 [file animals-16-00879-s001.zip › animals-4165716 - supplementary.pdf]

**Table S1.** Square table of Primer Reaction quantities of three species

| Primer of *E. Bieneusi* (*nmol*) | Primer of *Giardia lamblia* (*nmol*) | Primer of *Cryptosporidium*(*nmol*) |
|---|---|---|
| 0.002 | 0.002 | 0.002 |
| 0.002 | 0.002 | 0.004 |
| 0.002 | 0.002 | 0.006 |
| 0.002 | 0.004 | 0.002 |
| 0.002 | 0.004 | 0.004 |
| 0.002 | 0.004 | 0.006 |
| 0.002 | 0.006 | 0.002 |
| 0.002 | 0.006 | 0.004 |
| 0.002 | 0.006 | 0.006 |
| 0.004 | 0.002 | 0.002 |
| 0.004 | 0.002 | 0.004 |
| 0.004 | 0.002 | 0.006 |
| 0.004 | 0.004 | 0.002 |
| 0.004 | 0.004 | 0.004 |
| 0.004 | 0.004 | 0.006 |
| 0.004 | 0.006 | 0.002 |
| 0.004 | 0.006 | 0.004 |
| 0.004 | 0.006 | 0.006 |
| 0.006 | 0.002 | 0.002 |
| 0.006 | 0.002 | 0.004 |
| 0.006 | 0.002 | 0.006 |
| 0.006 | 0.004 | 0.002 |
| 0.006 | 0.004 | 0.004 |
| 0.006 | 0.004 | 0.006 |
| 0.006 | 0.006 | 0.002 |
| 0.006 | 0.006 | 0.004 |
| 0.006 | 0.006 | 0.006 |

**Table S2.** Square table of Probe Reaction quantities of three species

| Probe of *E. Bieneusi* (*nmol*) | Probe of *Giardia lamblia* (*nmol*) | Probe of *Cryptosporidium*(*nmol*) |
|---|---|---|
| 0.002 | 0.002 | 0.002 |
| 0.002 | 0.002 | 0.004 |
| 0.002 | 0.002 | 0.006 |
| 0.002 | 0.004 | 0.002 |
| 0.002 | 0.004 | 0.004 |
| 0.002 | 0.004 | 0.006 |
| 0.002 | 0.006 | 0.002 |
| 0.002 | 0.006 | 0.004 |
| 0.002 | 0.006 | 0.006 |
| 0.004 | 0.002 | 0.002 |
| 0.004 | 0.002 | 0.004 |
| 0.004 | 0.002 | 0.006 |
| 0.004 | 0.004 | 0.002 |
| 0.004 | 0.004 | 0.004 |
| 0.004 | 0.004 | 0.006 |
| 0.004 | 0.006 | 0.002 |
| 0.004 | 0.006 | 0.004 |
| 0.004 | 0.006 | 0.006 |
| 0.006 | 0.002 | 0.002 |
| 0.006 | 0.002 | 0.004 |
| 0.006 | 0.002 | 0.006 |
| 0.006 | 0.004 | 0.002 |
| 0.006 | 0.004 | 0.004 |
| 0.006 | 0.004 | 0.006 |
| 0.006 | 0.006 | 0.002 |
| 0.006 | 0.006 | 0.004 |
| 0.006 | 0.006 | 0.006 |
