# Supplementary material for: Development of Multiplex Real-Time Quantitative PCR for the Detection of Giardia duodenalis, Enterocytozoon bieneusi, and Cryptosporidium spp. in Dairy Goats
Source: Animals (Basel). 2026 Mar 11;16(6):879. doi: 10.3390/ani16060879 (PMC13023305; doi:10.3390/ani16060879)

## Slide 1
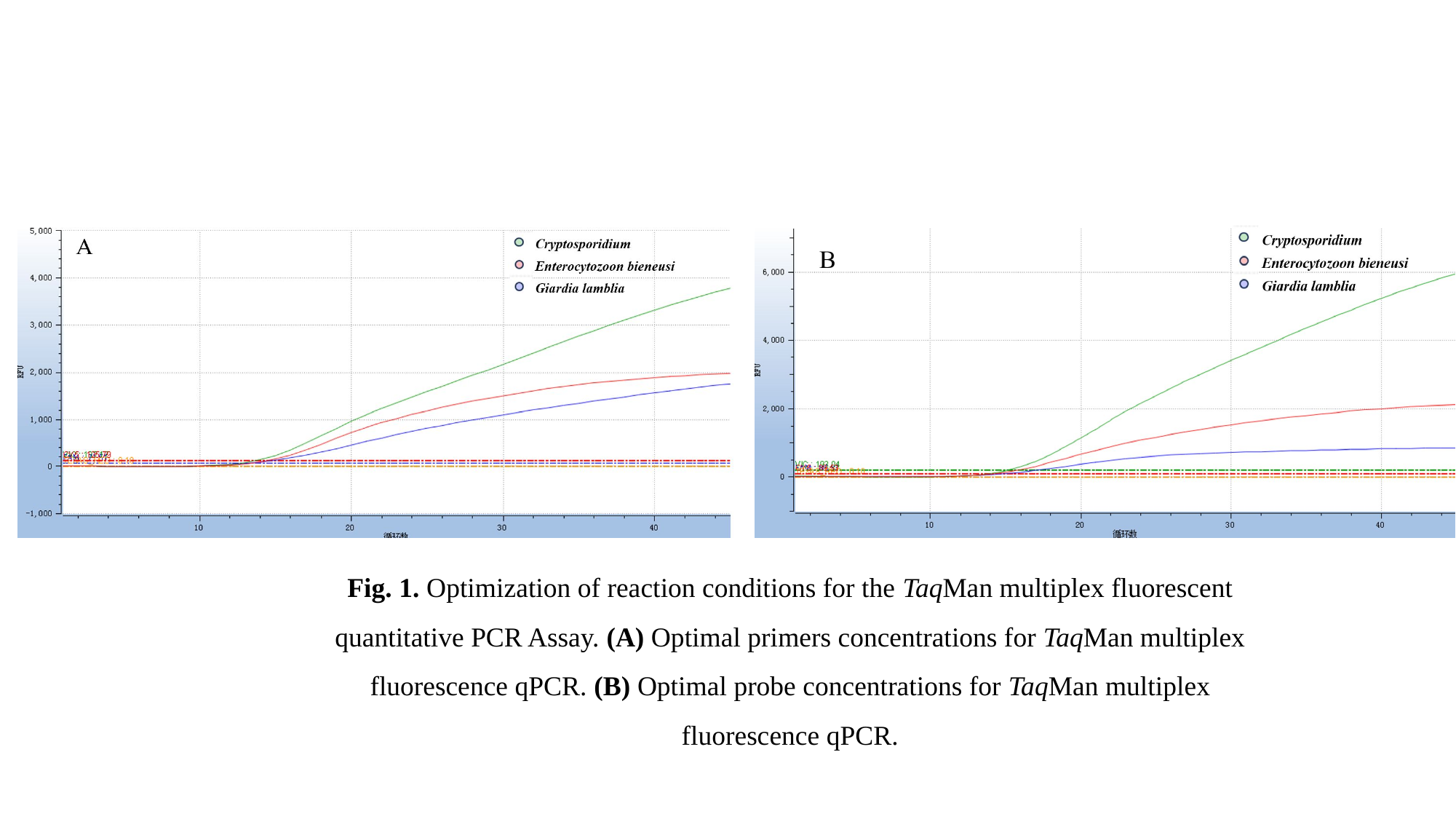

Fig. 1. Optimization of reaction conditions for the TaqMan multiplex fluorescent quantitative PCR Assay. (A) Optimal primers concentrations for TaqMan multiplex fluorescence qPCR. (B) Optimal probe concentrations for TaqMan multiplex fluorescence qPCR.

## Slide 2
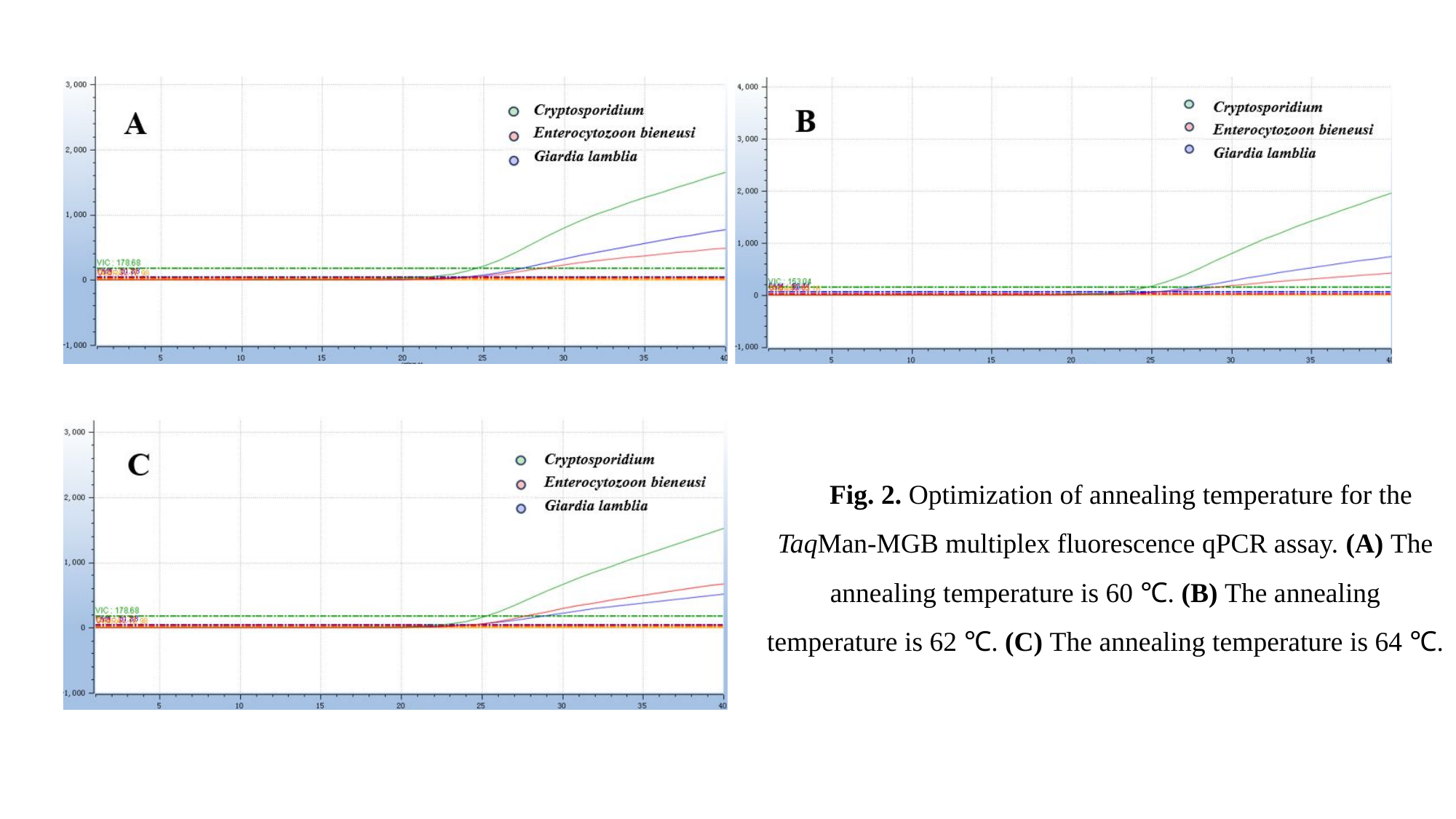

Fig. 2. Optimization of annealing temperature for the TaqMan-MGB multiplex fluorescence qPCR assay. (A) The annealing temperature is 60 ℃. (B) The annealing temperature is 62 ℃. (C) The annealing temperature is 64 ℃.

## Slide 3
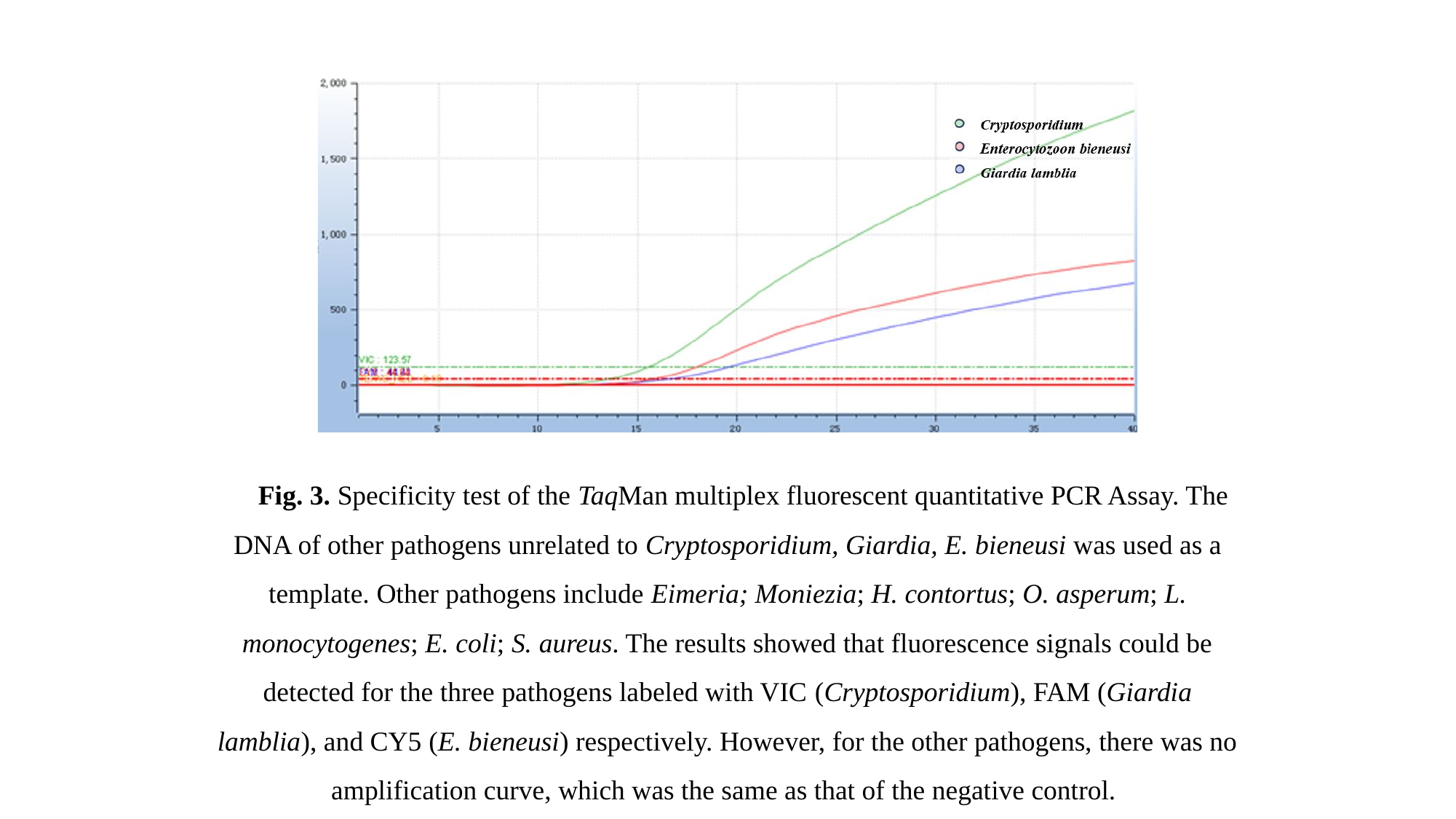

Fig. 3. Specificity test of the TaqMan multiplex fluorescent quantitative PCR Assay. The DNA of other pathogens unrelated to Cryptosporidium, Giardia, E. bieneusi was used as a template. Other pathogens include Eimeria; Moniezia; H. contortus; O. asperum; L. monocytogenes; E. coli; S. aureus. The results showed that fluorescence signals could be detected for the three pathogens labeled with VIC (Cryptosporidium), FAM (Giardia lamblia), and CY5 (E. bieneusi) respectively. However, for the other pathogens, there was no amplification curve, which was the same as that of the negative control.

## Slide 4
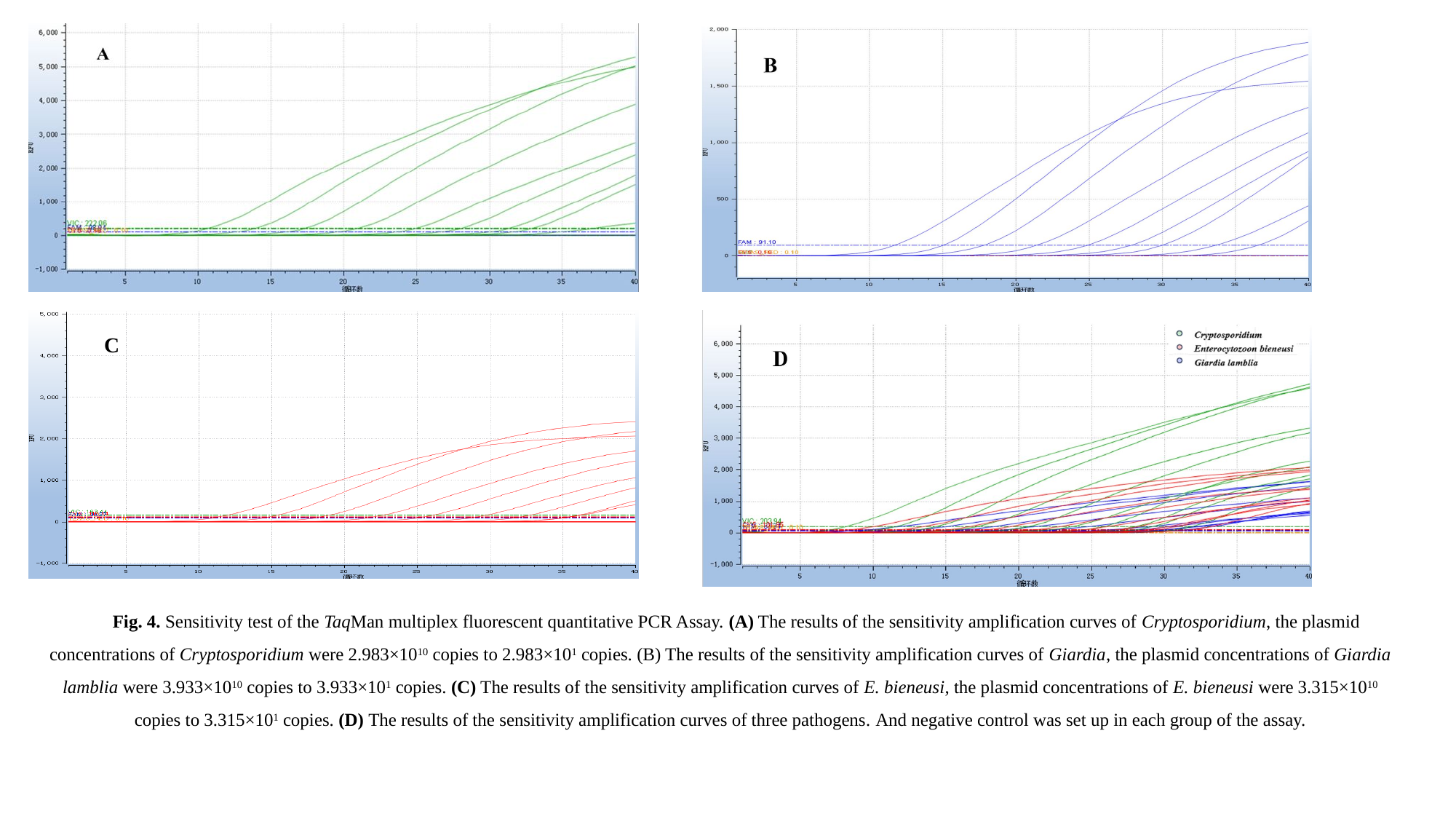

C
Fig. 4. Sensitivity test of the TaqMan multiplex fluorescent quantitative PCR Assay. (A) The results of the sensitivity amplification curves of Cryptosporidium, the plasmid concentrations of Cryptosporidium were 2.983×10^10^ copies to 2.983×101 copies. (B) The results of the sensitivity amplification curves of Giardia, the plasmid concentrations of Giardia lamblia were 3.933×10^10^ copies to 3.933×101 copies. (C) The results of the sensitivity amplification curves of E. bieneusi, the plasmid concentrations of E. bieneusi were 3.315×10^10^ copies to 3.315×101 copies. (D) The results of the sensitivity amplification curves of three pathogens. And negative control was set up in each group of the assay.

## Slide 5
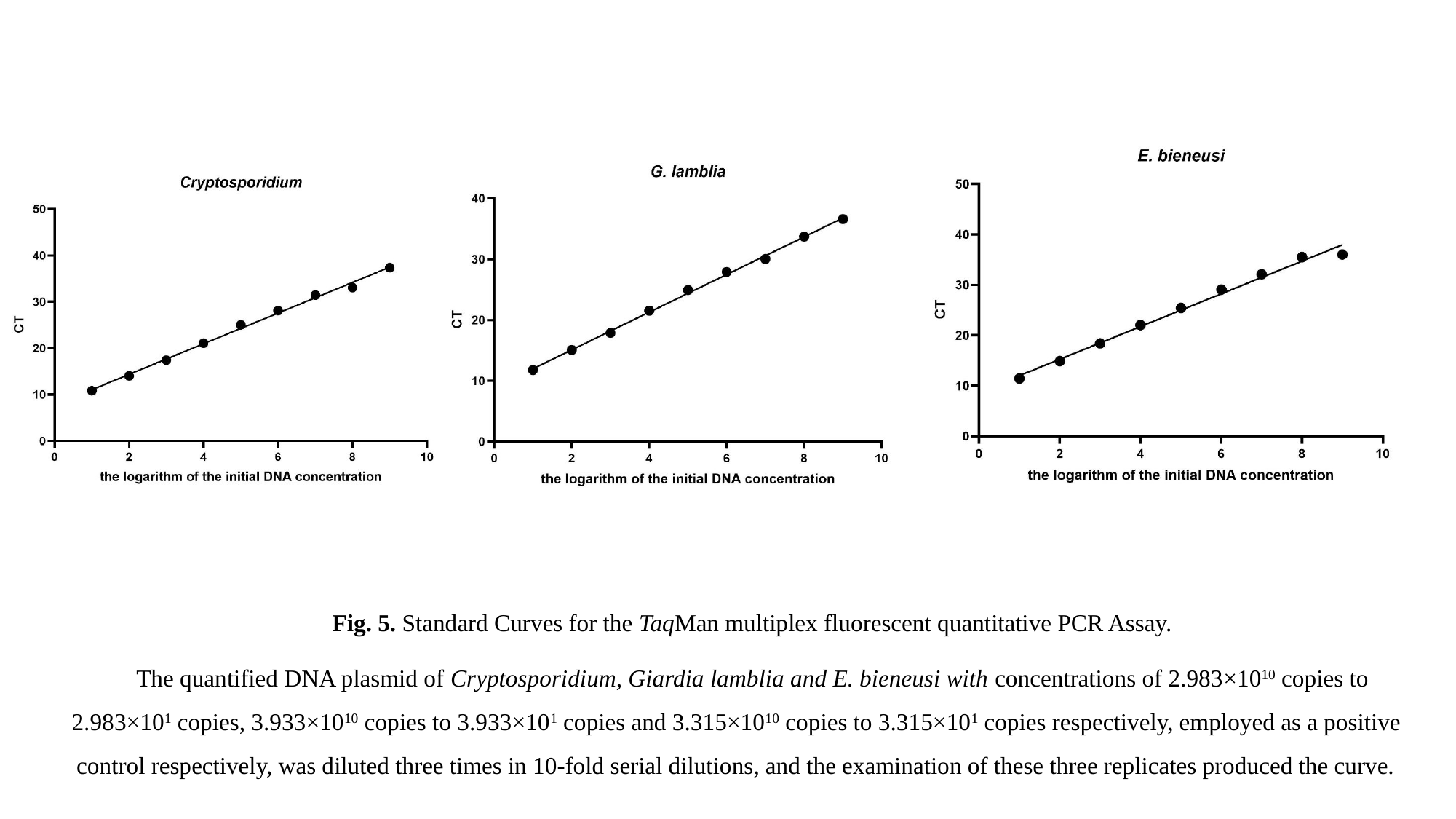

Fig. 5. Standard Curves for the TaqMan multiplex fluorescent quantitative PCR Assay.
The quantified DNA plasmid of Cryptosporidium, Giardia lamblia and E. bieneusi with concentrations of 2.983×10^10^ copies to 2.983×101 copies, 3.933×10^10^ copies to 3.933×101 copies and 3.315×10^10^ copies to 3.315×101 copies respectively, employed as a positive control respectively, was diluted three times in 10-fold serial dilutions, and the examination of these three replicates produced the curve.

## Slide 6
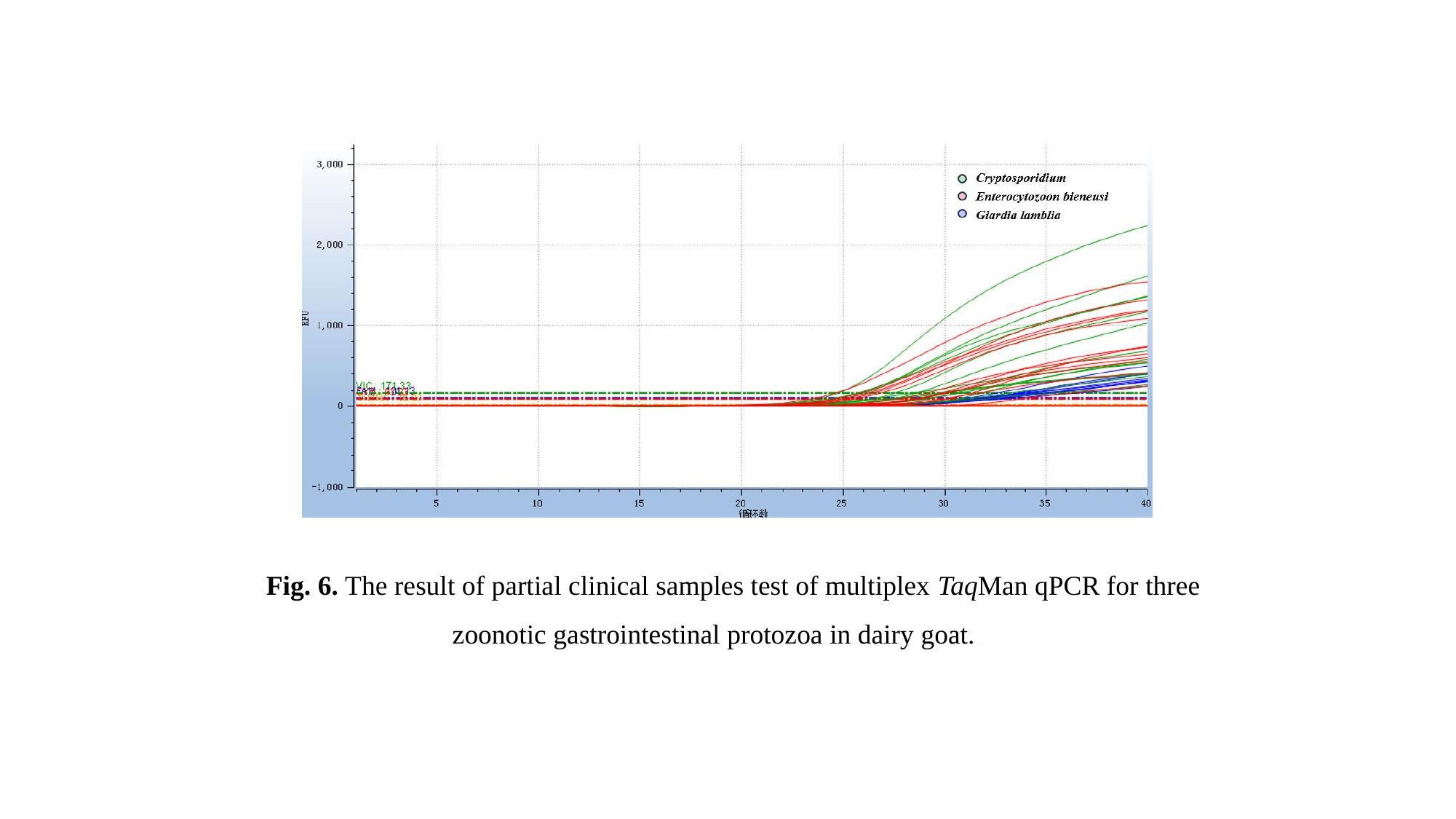

Fig. 6. The result of partial clinical samples test of multiplex TaqMan qPCR for three zoonotic gastrointestinal protozoa in dairy goat.

## Slide 7
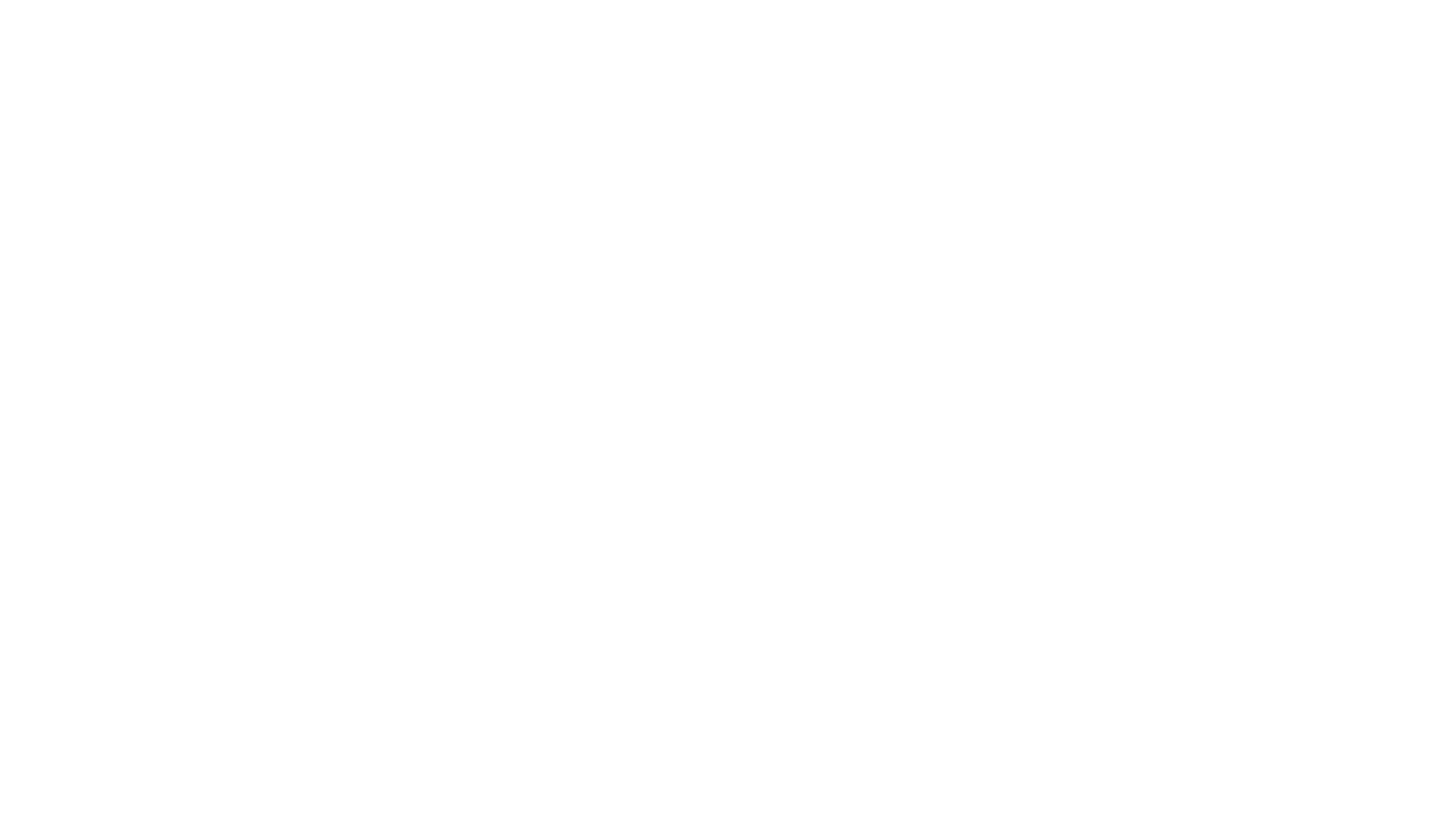

## Slide 8
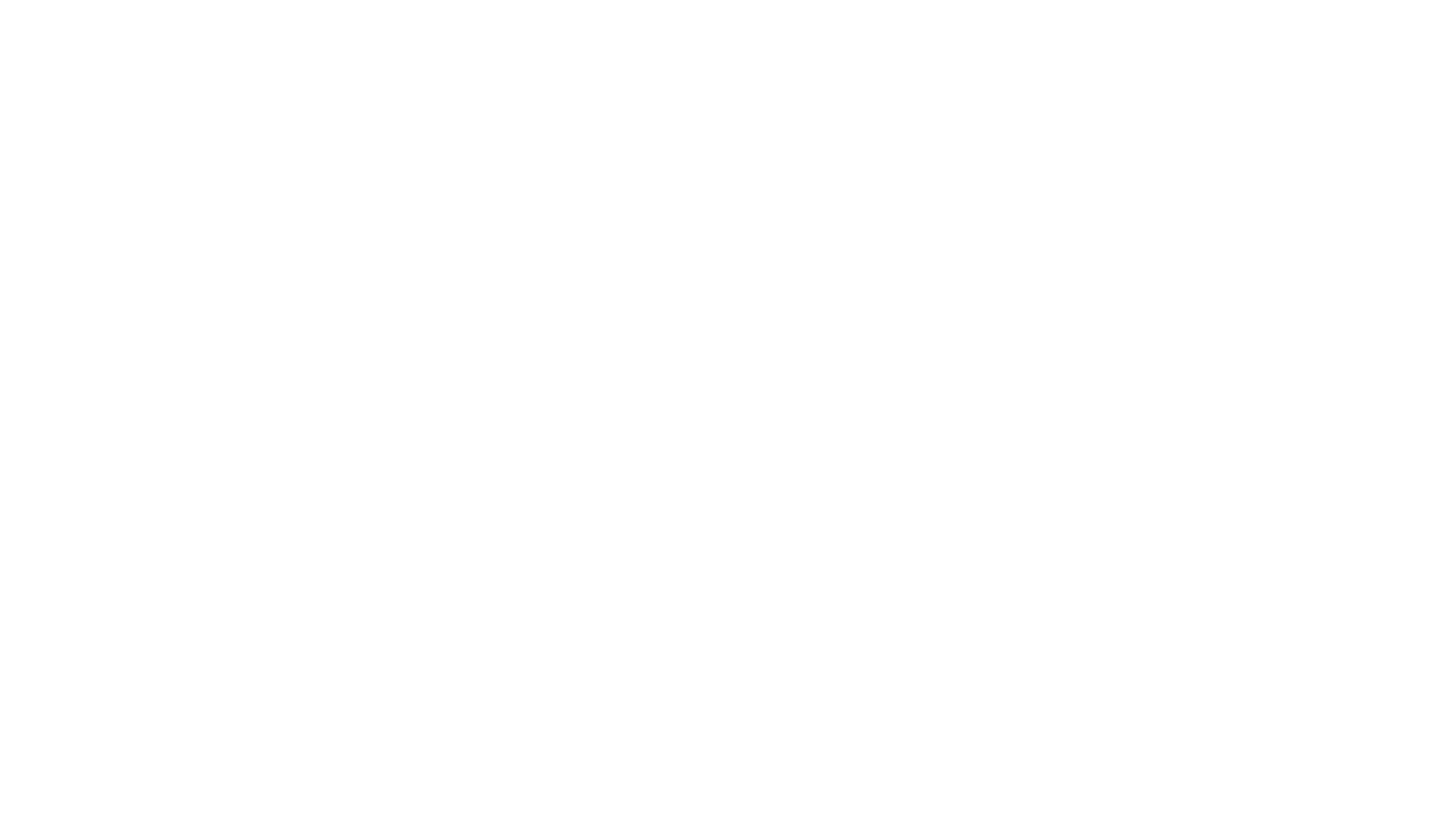

Supplement: Supplementary file 1 [file animals-16-00879-s001.zip › figures.pptx]
